# Inflammatory myofibroblastic tumor of the urinary bladder in a patient with the left renal cell carcinoma: A case report

**DOI:** 10.3892/etm.2014.1525

**Published:** 2014-02-07

**Authors:** SHUIQING WU, LE CHEN, QI WAN, LEI ZHANG, XIAOKUN ZHAO, XIUYING TANG

**Affiliations:** 1 Department of Urology, The Second Xiangya Hospital of Central South University, Changsha, Hunan 410011, P.R. China; 2 Department of Urology, Yueyang People’s Hospital Affiliated to Central South University, Yueyang, Hunan 414000, P.R. China

**Keywords:** bladder tumor, inflammatory myofibroblastic tumor, renal cell carcinoma

## Abstract

This case report describes an inflammatory tumor of the urinary bladder along with left renal cell carcinoma which occurred in a 73-year-old male with a narrowed orificium urethrae internum and severe hyperplasia of the prostate gland. A biopsy was not obtained prior to surgery. An inflammatory myofibroblastic tumor (IMT) of the urinary bladder is a rare benign lesion, particularly in the elderly. To the best of our knowledge, there are no studies concerning IMTs of the urinary bladder in patients with unilateral renal cell carcinoma. A bladder lesion due to an IMT may be easily misdiagnosed as metastasis from left renal cell carcinoma due to the hypervascularity of the tumor. In this case, radical surgery of the cancer of the left kidney was performed by laparoscopy. Subsequently, after three weeks and according to the examination of the intraoperative frozen-sections, a partial cystectomy was performed. Thus, radical resection of the bladder and the associated complications were avoided.

## Introduction

The inflammatory myofibroblastic tumor (IMT) of the urinary bladder is a rare benign lesion, particularly for the aged. To the best of our knowledge, there is no study about inflammatory myofibroblastic tumor of the urinary bladder in a patient with unilateral renal cell carcinoma. The bladder lesion may be easily masqueraded as metastasis from the left renal cell carcinoma due to its hypervascularity. First, we performed radical operation of the left kidney cancer with laparoscope, then after 3 weeks, according to the intraoperative frozen section examinations, partial cystectomy was performed. Thus avoiding radical resection of the bladder and corresponding complications.

## Case report

A 73-year-old male with hypertension, mild diabetes and benign prostatic hyperplasia was admitted to The Second Xiangya Hospital (Changsha, China). The patient complained of a one-year history of dysuria and presented with frequent and urgent urination, which was exacerbated by urodynia without hematuria for the prior three months.

**Figure 1:**
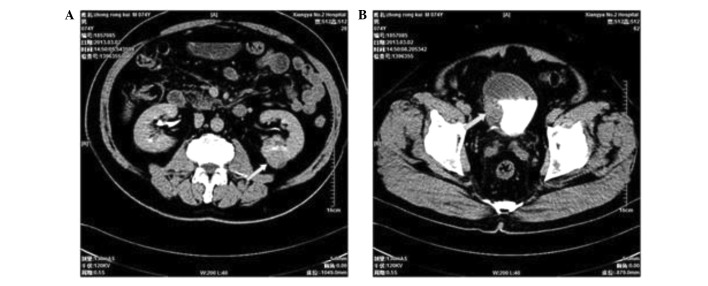
(A) Contrast-enhanced CT of the abdomen and pelvis of the patient showed an enhancing mass (arrow) in the left of the kidney. (B) Delayed-phase CT of the pelvis of the patient showed a mass of homogeneous low attenuation in the right and front walls of the bladder (arrow). CT, computerised tomography.

A computerised tomography (CT) scan of the patient was performed and reviewed. A 2.5-cm slightly enhancing mass was observed in the interpolar region of the left kidney and a 3×2-cm enhancing mass was observed in the right and front walls of the bladder (Fig. 1). A biopsy was not obtained prior to surgery. A positron emission tomography (PET)-CT scan was subsequently conducted and it revealed a 2.5×3.5-cm slightly enhancing mass, with partially abnormal PET detection in the interpolar region of the left kidney [maximum standardized uptake value (SUV), 2.5] and in the right and front wall of the bladder (maximum SUV, 3.5).

**Figure 2:**
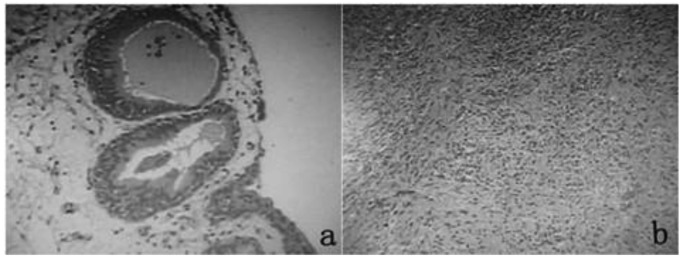
Microscopic findings: (A) Clear renal cell carcinoma (the left kidney); and (B) the visual field of the IMT is filled with numerous spindle cells and dispersal inflammatory cell infiltration (bladder tumor). IMT, inflammatory myofibroblastic tumor.

Under the diagnosis of left renal cell carcinoma with possible metastasis to the urinary bladder, surgery was performed. Laparoscopic exploration and radical resection of the left kidney were performed, while attempting to resect as much of the left ureter as possible. After the resection of the left kidney, the left renal tissue was transported to The Department of Pathology. The tissue was embedded in paraffin and sectioned by the doctors of The Department of Pathology in The Second Xiangya Hospital of Central South University (Changsha, China). The pathological results were published by the professors of The Department of Pathology in The Second Xiangya Hospital of Central South University. The resected left renal material was paraffin embedded and sectioned, and showed clear renal cell carcinoma (Fig. 2). Exploration of the bladder was performed three weeks later and a hard tumor of ~3×2 cm was identified. The tumor was festering partially, penetrating the serosa of the bladder and adhering to the surrounding tissues, and was palpable where the right side and front walls of the bladder merge. The mass was infiltrating the fat around the bladder, and the internal orifice of urethra was clearly constrictive. In our opinion, severe hyperplasia of the prostate gland and the severe inflammation of the bladder may have contributed to the stricture. The festering sections of the tumor were sent for rapid cross-section frozen section examination and the presence of cystitis glandularis was reported. Subsequently, under the impression that the bladder tumor was maligant, the entire tumor and the thickened parts of the bladder were sent to The Department of Pathology in The Second Xiangya Hospital of Central South University for rapid frozen cross-section examination and they indicated a low-grade malignant mesenchymal tumor. Ultimately, partial cystectomy was performed.

## Discussion

An IMT was first reported by Bahadori and Liebow in a lung lesion in 1973 (1). Tumor invasion sites were reported to be mainly in the lung and relatively rare in the urinary system. The tumor was subsequently termed a plasma cell granuloma of the lung and Yamamoto *et al* (2) reported that the occurrence of IMTs was associated with the expression levels of the genes p53 and murine double minute 2. In 2002, the World Health Organization (3) officially declared that IMTs were a type of mesenchymal tumor, few of which relapse or transfer.

IMTs develop at any age, but commonly arise in children and young adults, particularly in female individuals. The occurrence of IMTs in elderly patients (>70 years old) and a previous study (4) associated with IMTs combined with the unilateral renal clear cell carcinoma are extremely rare. In patients with an IMT of the urinary bladder with renal cell carcinoma, it may be difficult to differentiate the bladder lesion caused by the bladder cell metastasis from the renal cell carcinoma (4). The pathogenesis of IMTs is not clear and they present inflammatory cell infiltration in histopathological analysis. Chronic infection has been regarded as an important factor in the pathogenesis of IMTs, and the microorganisms that have been isolated from IMT lesions include mycobacteria, corynebacterium, Epstein-Barr virus and human papilloma virus (5). In the present case, the presence of diabetes and incomplete obstruction of the bladder outlet may have induced a chronic infection of the bladder of the patient.

The common clinical manifestations of IMTs of the bladder in hematological analysis include gross hematuria, an increased erythrocyte sedimentation rate, anemia and hypergammaglobulinemia (6). A small number of patients with an IMT present with frequent micturition, urgency of urination, odynuria and dysuria, lower abdominal pain or urinary tract symptoms (7) IMTs are usually reported by CT scanning as a 2- to 11-cm tumor in the bladder or submucosa (8), occasionally accompanied by fat infiltration around the bladder. In the majority of cases of IMTs, the imaging and cystoscopic findings are non-specific with its hypervascularity and invasiveness. The imaging and cystoscopic findings of this tumor always indicate the malignant tumors of the urinary bladder due to its non-specificity, IMTs of the bladder are difficult to diagnose, particularly prior to surgery. The bladder lesions caused by an IMT (4) are always locally aggressive and mimic those of malignancies, and it is easy to misdiagnose an IMT as a malignant bladder tumor. Furthermore, six months after treatment, patients with an IMT exhibit no recurrence.

Radical resection is the preferred method of treatment for IMTs of the bladder (9). The choice of partial resection of the bladder or transurethral resection of a bladder tumor depends on the depth of the tumor invasion. For large tumor masses, treatment of the patients with celecoxib and prednisone is attempted first (10), and then partial resection of bladder is performed if the tumor has narrowed.

In the present case, it was difficult to distinguish whether the two cases were due to transference as all the iconographic data, including the PET-CT scan, indicated that the masses in the left kidney and bladder of the patient may be malignant. Intraoperative rapidly frozen cross-section examination of IMT resection material is essential, particularly in the cases when it is not possible to collect a preoperative biopsy, in order to avoid radical resection of the bladder and the associated complications.
